# Leukotriene Production Is Increased in Abdominal Obesity

**DOI:** 10.1371/journal.pone.0104593

**Published:** 2014-12-01

**Authors:** Magnus Bäck, Antoine Avignon, Françoise Stanke-Labesque, Catherine Boegner, Vincent Attalin, Elodie Leprieur, Ariane Sultan

**Affiliations:** 1 Department of Medicine, Karolinska Institutet, Stockholm, Sweden; 2 Endocrinology-Diabetology-Nutrition Department, CHRU Montpellier, Montpellier, France; 3 U1046, INSERM, Université Montpellier 1, Montpellier, France; 4 INSERM U1042, Université de Grenoble, Grenoble, France; INSERM/UMR 1048, France

## Abstract

Obesity is a major risk factor for insulin resistance and type-2 diabetes. A chronic low grade inflammatory state has been described during obesity and associated with insulin resistance pathogenesis. Results from animal studies are in favor of a role of the leukotriene (LT) pathway in obesity induced-insulin resistance. However, there is a paucity of data regarding this association in human obesity. Therefore, the aim of this study was to investigate whether LT production was associated with insulin resistance and other metabolic parameters in a cohort of obese subjects. Forty-six (70% females) obese subjects (BMI≧30 kg/m^2^) without known diabetes and without inflammatory disease (CRP<10 mg/l) were included. Median age was 44 years (16–80) with a median BMI of 36.8 kg/m^2^ (30–51). Insulin resistance was evaluated by HOMA-IR index and glucose tolerance test. Urinary LTE_4_ (U-LTE_4_) concentration was measured by enzyme immune assay. Screening for obstructive sleep apnea was performed. There was a positive association of U-LTE_4_ with waist to hip ratio, systolic blood pressure and HOMA-IR in univariate analysis. Further, waist to hip ratio remained the only parameter significantly correlated with U-LTE_4_, in adjusted multivariate analysis. Taken together, these results confirm the previously established notion of chronic low grade inflammation in obesity and further suggests a role for the LT pathway in obesity-associated development of insulin resistance in humans.

## Introduction

Obesity is a major risk factor for insulin resistance and type-2 diabetes. A wealth of evidence indicates that these metabolic comorbidities are associated with presence of low-grade chronic inflammation. Indeed, in adipose tissue, the production of proinflammatory cytokines is increased, accompanied by a reduction in the anti-inflammatory and insulin-sensitizing adiponectin [1],[2]. In addition to adipokines, adipose tissue inflammation may also be driven by activation of classical proinflammatory pathways such as arachidonate lipoxygenases, leading to the formation of leukotrienes (LT) [3]–[5]. Indeed, adipose tissue overexpression of selected members of the 5-lipoxygenase pathway and increased LT production have been recently described in animal models [3],[6], including obese rodents [3],[6],[7]. In these conditions, 5-lipoxygenase products exert potent proinflammatory actions, such as induction of nuclear factor (NF)-κB [8] and secretion of proinflammatory and insulin resistant adipokines (i.e., monocyte chemotactic protein-1, tumor necrosis factor-α, macrophage inflammatory protein-1, and interleukin-6) that could potentially contribute to systemic insulin resistance. Furthermore, the physiological consequences of these changes in adipose tissue function were corroborated *in vivo* by the observation that inhibition of the 5-lipoxygenase pathway reduced proinflammatory cytokines [3],[6] and circulating free fatty acid concentrations [7], as well as alleviated insulin resistance [6],[9] and hepatic steatosis in experimental obesity [10]. However, there is a paucity of information on the association of the LT pathway with insulin resistance derived from human studies.

In humans, the association between LT production and obesity was initially demonstrated in subjects with obstructive sleep apnea syndrome (OSAS) [11], and was subsequently reported in asthmatics [12] and in children with sleep disordered breathing [13]. In addition, the 5-lipoxygenase activating protein (FLAP) is expressed in human adipose tissue, with higher levels in abdominal subcutaneous fat derived from obese compared with lean subjects [14]. One of the possible mechanism linking obesity and asthma concerns immunoregulation and inflammation. For example, urinary levels of leukotriene E_4_ (U-LTE_4_) is a validated biomarker of asthma [8], and also increases gradually with body mass index (BMI) [11]. However, the risk of asthma due to obesity remains unclear and attempts to investigate the influence of obesity on asthmatic airway and systemic inflammation have generated conflicting results [15]–[17], and the association between these two conditions has been questioned [18],[19]. Nevertheless, enhancement of adipose tissue functions in obesity could potentially lead to a pro-inflammatory state, which propagates either airway or systemic inflammation (or both) through the release of inflammatory variables [20],[21], such as LT. In support of the latter notion, leukotriene receptor antagonism provides its best disease control in obese asthmatics [22].

No previous study has however explored LT production during obesity and its relationship with insulin resistance in humans, independently of any asthma. Therefore, the aim of the present study was to investigate whether LT production was associated with insulin resistance and with metabolic parameters in a cohort of obese subjects.

## Materials and Methods

### Study population

Subjects admitted in the University Hospital of Montpellier (France) for assessment of obesity (BMI≧30 kg/m^2^) between January 2011 and February 2012 were included. Exclusion criteria were treatment with anti-inflammatory medications, asthma, known diabetes mellitus (hemoglobin A1c HbA1c >6,5%; fasting plasma glucose >7 mmol/l and/or treatment with any anti-diabetic medications), and hs-CRP above 10 mg/l. All participants had normal cardiopulmonary functions as determined by medical history, physical examination and electrocardiogram. Height and weight were measured wearing light clothing and no shoes. BMI was calculated as weight in kg divided by the square of height in meters (kg/m^2^). Waist circumference was recorded to the nearest 0.1 cm midway between the last rib and the iliac crest using a non-stretch tape measure on lightly clad participants.

All clinical investigations were conducted according to the principles expressed in the Declaration of Helsinki and in compliance with International Conference on Harmonization/Good Clinical Practice regulations. According to the French Law, the study has been registered at “Ministère de la Santé et de l'Enseignement Supérieur” after approval by the Montpellier University Hospital's ethics committee (Comité de Protection des Personnes Sud Méditerranée IV) with the following number DC-2009-1052. All patients gave their written informed consent. For minor participants, written informed consent were obtained from the next of kin, following ethics committee recommendations.

### Sleep screening

Sleep monitoring was performed in 46 subjects using Alice PDx (Philips). Sleep apnea was defined as apnea-hypopnea index (AHI) >5 per hour of sleep. OSA was considered mild (5>AHI<15), moderate (15≧AHI<30), or severe (≧30).

### Laboratory procedures

Blood was drawn in the morning after an 8 h overnight fast for the following serum measurements: Fasting plasma glucose (FPG), HbA1c was analyzed by routine high-performance liquid chromatography (HPLC)-based ion-exchange procedure (HA-8140; Menarini, Rungis Cedex, France). Total cholesterol, triglycerides (TG) high density lipoprotein and creatinine were measured using routine enzymatic methods (KonePro; Konelab, Epoo, Finland). Hs-CRP was assessed by immunoturbidimetry (Randox reagents on Olympus apparatus, Rungis, France).

The Cockroft-Gault formula was calculated utilizing the ideal body weight to estimate glomerular filtration rate (eGFR). LDL cholesterol was calculated according to the Friedwald formula. The Homeostatic Model Assessment index-insulin resistance (HOMA-IR) was calculated as follows: fasting insulin×fasting glucose/22.5 (insulin-resistance was defined by a HOMA-IR ≧3) [23]. Since nearly all subjects were characterized by an increased in waist circumference, above the diagnosis threshold for metabolic syndrome [24], we did not define metabolic syndrome in the present study, to avoid selection bias. Abdominal obesity was defined according to the waist to hip ratio as described by the World Health Organization in order to be more discriminant [25]. Consequently, metabolic parameters were defined by the following cut-offs: waist to hip ratio >0.9 (men) >0.85 (women); TG >1.7 mmol/L; HDL<1.0 mmol/L (men)<1.3 mmol/L (women); hypertension (systolic blood pressure >130 mmHg, diastolic arterial pressure >85 mmHg or anti-hypertensive treatment, high fasting blood glucose (>5 mmol/L).

Urine was collected in the morning and frozen in aliquots at −80°C until analysis. U-LTE_4_ was measured using enzyme immune assay kits from Cayman Chemicals (Ann Arbor). Urinary creatinine was determined using a standard colorimetric assay.

### Statistical analysis

Data are expressed as median (range) and frequencies are expressed as percentages. A nonparametric Mann-Whitney Rank Sum Test was used for comparisons between 2 groups. A two-way analysis of variance (ANOVA) test was used to compare the clinical and biochemical characteristics of the cohort (Table 1). Correlations between the urinary-LTE_4_ and metabolic parameters were established by Spearman correlation. A multiple stepwise linear regression was performed to evaluate the impact of the components of the metabolic syndrome on U-LTE_4_. *P*<0.05 was considered significant. Analyses were performed using SigmaPlot version 12 (Systat Software Inc).

**Table 1:** Clinical and biochemical characteristics of the patient cohort (N = 46) stratified according to sex.

Characteristics	All (n = 46)	Males (n = 14)	Females (n = 32)	*P*
**Age (years)**	44 (16–80)	43.5 (16–60)	44 (22–80)	0.47
**Weight (kg)**	105 (64–150)	111 (95–150)	96 (64–140)	0.009
**BMI (kg/m^2^)**	36.8 (30–51)	36.3 (31–48)	37.0 (30–51)	0.99
**Waist (cm)**	111 (81–150)	119 (103–142)	109 (81–150)	0.009
**Waist to hip ratio**	0.89 (0.72–1.13)	0.98 (0.84–1.13)	0.86 (0.72–1.0)	<0.001
**Systolic blood pressure (mmHg)**	122 (97–170)	128 (110–140)	120 (97–170)	0.14
**Diastolic blood pressure (mmHg)**	72 (55–93)	72 (68–93)	71 (55–86)	0.32
**Total cholesterol (mmol/L)**	5.4 (2.8–7.3)	5.4 (3.1–7.3)	5.2 (2.8–7.3)	0.93
**LDL cholesterol (mmol/L)**	3.4 (1.1–4.9)	3.4 (1.1–4.4)	3.4 (1.8–4.9)	0.47
**HDL cholesterol (mmol/L)**	1.14 (0.73–1.8)	1.09 (0.73–1.8)	1.24 (0.85–1.7)	0.020
**Triglycerides (mmol/L)**	1.6 (0.72–3.5)	2.1 (0.99–3.5)	1.5 (0.72–3.1)	0.010
**HOMA-IR**	2.58 (1.34–13)	2.92 (1.34–13)	2.57 (1.43–8.54)	0.23
**Hb1Ac (%)**	5.8 (4.9–7.0)	5.8 (4.9–6.8)	5.9 (5.1–7.0)	0.17
**Fasting glucose (mmol/L)**	4.9 (4.1–6.9)	4.9 (4.1–6.6)	5.0 (4.1–6.9)	0.55
**Fasting insulin (mU/L)**	12.5 (6.6–43.7)	12.1 (7.0–24.8)	13.6 (6.6–43.7)	0.026
**WBC (1000/m^3^)**	6.7 (0.4–10.9)	6.8 (0.4–10.9)	6.4 (4.2–9.6)	0.15
**P-Creatinine** (µM)	64 (42–103)	62 (42–86)	78 (44–103)	0.000
**U-Creatinine** (mM)	6.8 (5.6–19)	7.2 (3.2–14)	4.9 (1.5–18)	<0.001
**eGFR** (mL/min)	93 (42–120)	87 (42–119)	100 (79–120)	0.09

Data are expressed as median (range).

## Results

### Patient characteristics

Fifty nine subjects were included after the initial screen. Of these, 11 had CRP-levels over 10 mg/L (defined as exclusion criteria, *cf.* above). Another two subjects were excluded because of non-usable urine samples. The clinical and biochemical characteristics of the remaining 46 subjects (70% females) of the study cohort are shown in Table 1.

### U-LTE_4_ in relation to metabolic parameters

**Figure 1:**
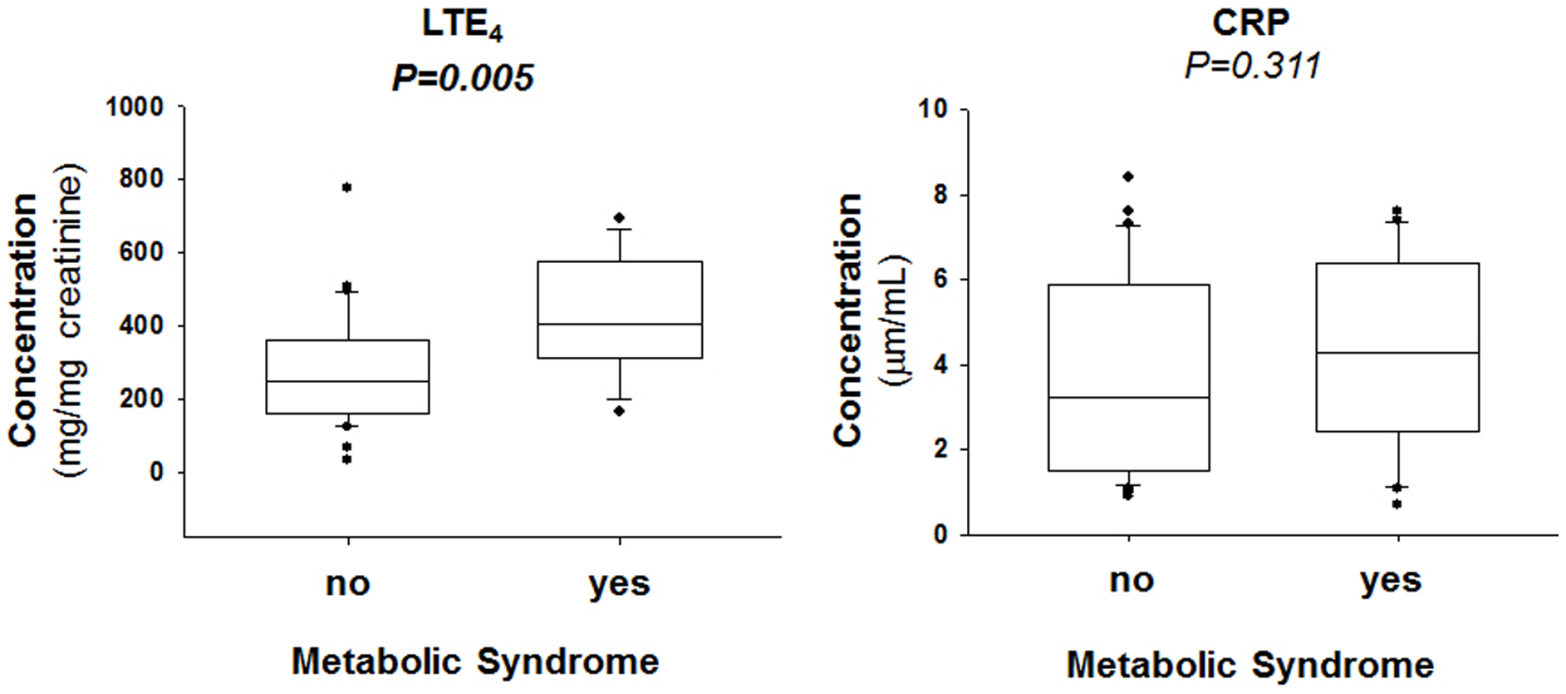
Urinary leukotriene E_4_ (LTE_4_; upper panels) and serum C-reactive protein (CRP; lower panels). Waist/Hip Ratio was categorized as either low (*n* = 19) or high (men>0.9; women>0.85; *n* = 26). Triglyceride levels were categorized as either low (*n* = 26) or high (>1.7 mmol/L; *n* = 20). HDL levels were categorized as either high (*n* = 22) or low (<1.0 mmol/L for men, and <1.3 mmol/L for women; *n* = 24). Hypertension was defined as systolic blood pressure >130 mmHg, mean arterial pressure >85 mmHg or anti-hypertensive treatment (*n* = 19). Fasting blood glucose was categorized as either low (*n* = 38) or high (>5 mmol/L; *n* = 8). *P*-value (obtained by means of Mann-Whitney Rank Sum Test) in bold font indicates *P*<0.05.

To assess what metabolic parameters predicted U-LTE_4_, a stratified comparison was performed for each factor, as shown in Fig. 1. Whereas the levels of U-LTE_4_ did not differ in groups with and without hypertension, and not between high and low HDL and TG, U-LTE_4_ was significantly higher in subjects with a high waist to hip ratio and in subjects with high fasting plasma glucose (Fig. 1). Although there was a tendency for a higher CRP in subjects with a high waist to hip ratio, none of the groups significantly differed in terms of CRP levels (Fig. 1).

### Univariate analyses

The univariate analyses for U-LTE_4_ with these factors as continuous variables are shown in Table 2. To extend the findings for waist circumference, also other anthropometric variables (BMI, weight) were included in the univariate analysis. Likewise, to extend the findings on fasting blood glucose (Fig. 1), also HOMA-IR index was calculated and examined for correlations with U-LTE_4_, as well as Hb1Ac. Furthermore, the full lipid profile was examined in relation to U-LTE_4_ and confirmed that there were no significant associations (Table 2). Finally, there was no significant correlation between U-LTE_4_ and renal function, estimated as GFR calculated using the ideal body weight (Table 2). Interestingly, univariate analysis revealed significant associations of U-LTE_4_ with waist to hip ratio, systolic blood pressure and HOMA-IR index.

**Table 2:** Univariate Spearman correlations between U-LTE_4_ and clinical/biological parameters (*n* = 46).

	R	P
Waist to Hip Ratio	0.371	**0.014**
Waist Circumference	0.261	0.083
Hip Circumference	0.0913	0.554
Weight	0.155	0.308
BMI	0.104	0.494
Cholesterol	−0.0171	0.91
LDL	0.00936	0.951
HDL	−0.128	0.401
TG	−0.00086	0.995
Systolic Blood Pressure	0.295	**0.049**
Diastolic Blood Pressure	0.14	0.358
HOMA-IR	0.393	**0.0086**
HbA1c	0.221	0.143
Fasting blood glucose	0.125	0.411
eGFR	0.218	0.218

### Multivariate analysis

An age- and gender-adjusted multivariate analysis including the waist to hip ratio, BMI, LDL level, HDL and TG level, blood pressure, HOMA-IR, and Hb1Ac revealed that the waist to hip ratio remained the only parameter significantly correlated with U-LTE_4_ (*P* = 0.015). This association remained significant (*P* = 0.023) after addition of eGFR to the multivariate analysis.

### U-LTE_4_ in relation to sleep apnea

In further search for potential confounders for the observed association of U-LTE_4_ with abdominal obesity and decreased glucose resistance, and since the association of U-LTE_4_ with obstructive sleep apnea is well established, sleep monitoring was performed. Three patients were treated with Continuous Positive Airway Pressure (CPAP) and were therefore excluded from this analysis. Of the remaining 43 subjects, 25 subjects had sleep apnea (defined as AHI>5), of which the majority (n = 16; 64%) has mild sleep apnea, whereas 16% (n = 4) had moderate and 20% (n = 5) severe sleep apnea. U-LTE_4_ did not differ between the groups with and without positive sleep apnea screening. In addition, univariate analyses did not reveal any significant correlations between U-LTE_4_ and the parameters obtained at polygraphy (AHI, mean SaO_2_, SaO_2_<90% in % of total sleep time). Furthermore, including also sleep parameters in the multivariate analysis above revealed that the waist to hip ratio remained an independent predictor of U-LTE_4_ also after adjustment for AHI.

### U-LTE_4_ in relation to hsCRP

Finally, we compared the predictive value of U-LTE_4_ with another established inflammatory marker, *i.e.* hsCRP. There was no significant correlation between hsCRP and U-LTE_4_ (*p* = 0.419). The waist to hip ratio remained an independent predictor for U-LTE_4_ also after adjusting for CRP. In addition, a similar multivariate age- and gender-adjusted analysis (including the waist to hip ratio, BMI, LDL, HDL, TG, blood pressure, HOMA-IR, and Hb1Ac) revealed BMI as the only significant correlate with hsCRP (*P*<0.001).

## Discussion

The association of U-LTE_4_ with metabolic parameters in the present cohort of obese subjects provides support to the notion that adipose tissue may serve as a source of inflammatory mediators interfering with insulin sensitivity. Interestingly, these associations were not observed for the established inflammatory marker CRP, and remained significant after adjustment for CRP. Taken together the results of the present study confirm the previously established notion of chronic low grade inflammation in obesity [26]–[28], and extend the observation by associating the 5-lipoxygenase pathway with metabolic parameters in obese subjects.

A previous study has associated U-LTE_4_ with type 1 diabetes [29]. Interestingly, intense glycemic control lowers systemic inflammatory parameters in type 1 diabetes, whereas inflammatory mediators including U-LTE_4_ remain unchanged before and after intense glycemic control in type 2 diabetes [30]. In the present study, group comparisons and univariate analyses suggested that fasting plasma glucose, waist to hip ratio and blood pressure may affect the U-LTE_4_ concentrations. However, a multivariate analysis identified that only the waist to hip ratio was an independent predictor of U-LTE_4_ concentrations. It is well demonstrated that accumulation of visceral fat is positively correlated with insulin resistance [31]. Visceral fat has some specific properties, such as adipokines and cytokines production as well as increased lipolysis, which may be deleterious on insulin sensitivity [32]. Therefore, both infiltrating immune cells and adipocytes in visceral adipose tissue may contribute to LT production and an aggravated chronic low grade inflammation [3],[6],[14].

Since this is an observational study, no definite conclusion of causality can be drawn from the present results. One possible mechanism is that increased LT production from visceral fat may be involved in the development of insulin resistance. This notion has received support from animal models, in which targeting either leukotriene receptor signaling or LT synthesis decreases proinflammatory cytokine secretion from visceral fat [3],[6] and protects against insulin resistance as a result of diet-induced obesity [6],[9]. In addition, glucose and insulin levels are lower in mice lacking a functional LT synthesis, hence supporting a crucial role of the LT pathway in metabolic control [6]. Further studies are needed to investigate the precise mechanisms involved in LT-induced metabolic effects in human disease. In this context, LT-induced effects on insulin signaling, lipolysis, adipogenesis and cytokine production, as well as systemic inflammation must be further explored. Moreover, it would be of particular importance to determine if LTs act in a paracrine and/or endocrine manner on other insulin sensitive tissues.

In contrast to previous studies [11],[13],[33], the present study did not reveal a significant association of the LT pathway with OSAS. However, previous analyses revealed that the influence of BMI on U-LTE_4_ concentrations was more than 3-fold more important compared with hypoxia severity [11]. Since the subjects in the present cohort were all obese and had substantially higher BMI (median 37 kg/m^2^), compared with the previous study cohort (median BMI 26 kg/m^2^) [11], the effect of obesity may have blunted the association of U-LTE_4_ with nocturnal hypoxia The latter notion is supported by the observation that CPAP treatment failed to reduce U-LTE_4_ in overweight and obese subjects with OSAS while it was efficient in normal weight OSAS patients [11]. Furthermore, although the previous study established the association of U-LTE_4_ with BMI, no significant association was obtained of within the present cohort of subjects with BMI≧30 kg/m^2^, further supporting that in obese subjects, the adipose tissue distribution (reflected by the significant association with the waist to hip ratio) rather than the BMI itself is the strongest determinant of U-LTE_4_ levels at BMI beyond 30 kg/m^2^. Methodological differences in LTE_4_ measurements between the present and the latter studies (ELISA vs. liquid chromatographic tandem mass spectrometry) should however also be acknowledged.

In the multivariate analysis, BMI was a significant predictor of CRP in the present study, which confirms previous studies [11]. In addition, previous studies in larger cohorts have demonstrated that CRP increases with the number of characteristics of metabolic syndrome, and is correlated with the cardiovascular outcome in these patients [34]. Importantly, the associations observed for U-LTE_4_ remained statistically significant also after adjustment for CRP, suggesting that U-LTE_4_ may have an additive value as biomarker in the context of abdominal obesity.

Although the present study provides a first indication of an association of the LT pathway with insulin resistance in the context of obesity in humans, several limitations must be acknowledged. First, this is a relatively limited group of obese patients. Nevertheless, the subjects were well characterized in terms of metabolic parameters, insulin resistance and associated sleep disordered breathing, hence providing the possibility to address several possible confounders. Furthermore, since nearly all the cohort had android obesity, the definition used in the present study replaced waist circumference by waist to hip ratio according to the WHO definition [24],[25], in order to increase the specificity of the observed associations. Second, the inflammatory parameters measured were limited to CRP and U-LTE_4_, and notably no cytokine concentrations were evaluated. Third, systemic evaluation of insulin sensitivity was performed using routine measurements, and further studies are needed to confirm these measures using gold standard technics, such as hyperglycemic and/or hyperinsulinemic clamp. Fourth, the present cohort lacked data on atherosclerosis, which is associated with both metabolic syndrome [35] and an up-regulation of the leukotriene pathway [33]. It can hence not be excluded that subclinical atherosclerosis may have contributed to the increased U-LTE_4_ in abdominal obesity in the present study. Finally, renal function should be considered when studying urinary biomarkers. It was for example recently shown that eGFR was an independent predictor of U-LTE_4_ in a cohort of diabetes patients with microalbuminuria and a median eGFR of 71 mL/min [36]. However, in the present cohort, the median eGFR (estimated using the ideal body weight) was within the normal range and did not significantly correlate with U-LTE_4_. Adjusting for this parameter did not change the multivariate analysis, suggesting that renal function was not a significant confounder in the present study.

In summary, the present study points to an association between the LT pathway and metabolic parameters in obese subjects, with the waist to hip ratio being an independent predictor of U-LTE_4_, suggesting visceral adipose tissue as determinant of LT production. In conclusion, these results may argue in favor of a role of the LT pathway in insulin resistance during obesity and need to be confirmed in larger studies, with analysis of the potential mechanisms involved. Nevertheless, these findings are quite encouraging from a clinical point of view, since anti-leukotriene treatment is currently used in clinical routine for asthma treatment. Interestingly, a recent study has in addition demonstrated beneficial effects of the leukotriene receptor antagonist montelukast on cardiovascular outcomes [37]. If our results are confirmed, it would be interesting to evaluate the effects of anti-leukotriene treatment on insulin resistance.
