# Correction: Leukotriene Production is Increased in Abdominal Obesity

**DOI:** 10.1371/journal.pone.0117861

**Published:** 2015-01-23

**Authors:** The PLOS ONE Staff

The image for Figure 1 is incorrect. Please see the corrected Figure 1 here.

**Figure 1:**
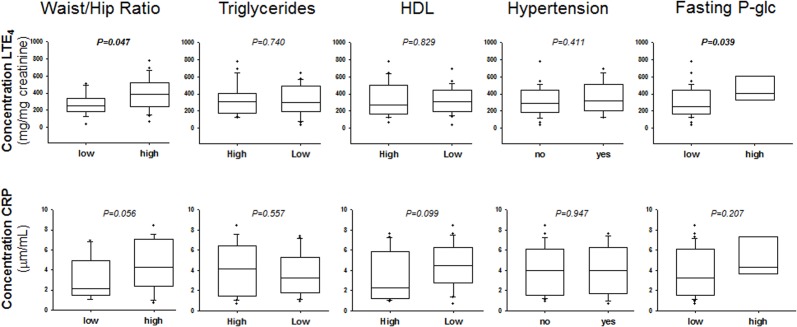
Urinary leukotriene E4 (LTE4; upper panels) and serum C-reactive protein (CRP; lower panels). Waist/Hip Ratio was categorized as either low (n = 19) or high (men>0.9; women>0.85; n = 26). Triglyceride levels were categorized as either low (n = 26) or high (>1.7 mmol/L; n = 20). HDL levels were categorized as either high (n = 22) or low (<1.0 mmol/L for men, and <1.3 mmol/L for women; n = 24). Hypertension was defined as systolic blood pressure >130 mmHg, mean arterial pressure >85 mmHg or anti-hypertensive treatment (n = 19). Fasting blood glucose was categorized as either low (n = 38) or high (>5 mmol/L; n = 8). P-value (obtained by means of Mann-Whitney Rank Sum Test) in bold font indicates P<0.05.
